# TETRASPANINs in Plants

**DOI:** 10.3389/fpls.2017.00545

**Published:** 2017-04-18

**Authors:** Ronny Reimann, Benedikt Kost, Jan Dettmer

**Affiliations:** Cell Biology, Biology Department, Friedrich-Alexander University Erlangen-NurembergErlangen, Germany

**Keywords:** tetraspanins, Arabidopsis, rice, plant reproduction, embryogenesis, plant growth

## Abstract

Tetraspanins are small transmembrane proteins that laterally associate with each other and cluster with numerous partner proteins as well as lipids. These interactions result in the formation of a distinct class of membrane domains, the tetraspanin-enriched microdomains (TEMs), which influence numerous cellular processes such as cell adhesion and fusion, intracellular membrane trafficking, signaling, morphogenesis, motility as well as interaction with pathogens and cancer development. The majority of information available about tetraspanins is based on studies using animal models or cell lines, but tetraspanins are also present in fungi and plants. Recent studies indicate that tetraspanins have important functions in plant development, reproduction and stress responses. Here we provide a brief summary of the current state of tetraspanin research in plants.

## Tetraspanin protein family

A family of membrane proteins largely overlooked in plants are the tetraspanins, small (200–350 aminoacids; Hemler, 2005) integral membrane proteins that are important in animals for cellular functions such as cell adhesion, fusion, polar growth, membrane trafficking, signaling, motility, and morphogenesis (Hemler, 2008; Yáñez-Mó et al., 2009). Tetraspanins were discovered in the early 1990s in immune cells, cancer cells and human parasites (Classon et al., 1990; Oren et al., 1990; Wright et al., 1990; Maecker et al., 1997). Since then tetraspanins have been identified in various eukaryotes including social amoeba (*Dictyostelium discoideum*, at least 5 tetraspanins), fungi (*Magnaporthe grisea*, a plant pathogen, at least 2 tetraspanins), plants (*Arabidopsis thaliana*, 17 tetraspanins), animals (*Drosophila melanogaster* 37 tetraspanins) and humans (33 tetraspanins) (Gourgues et al., 2002; Huang et al., 2005; Lambou et al., 2008; Boavida et al., 2013; Charrin et al., 2014). Despite their involvement in various cellular and developmental processes and their implication in diseases or pathologies, the molecular function of tetraspanins is still far from being understood (Potel et al., 2013; Halova and Draber, 2016).

## Tetraspanin structure and post translational modifications

**Figure 1:**
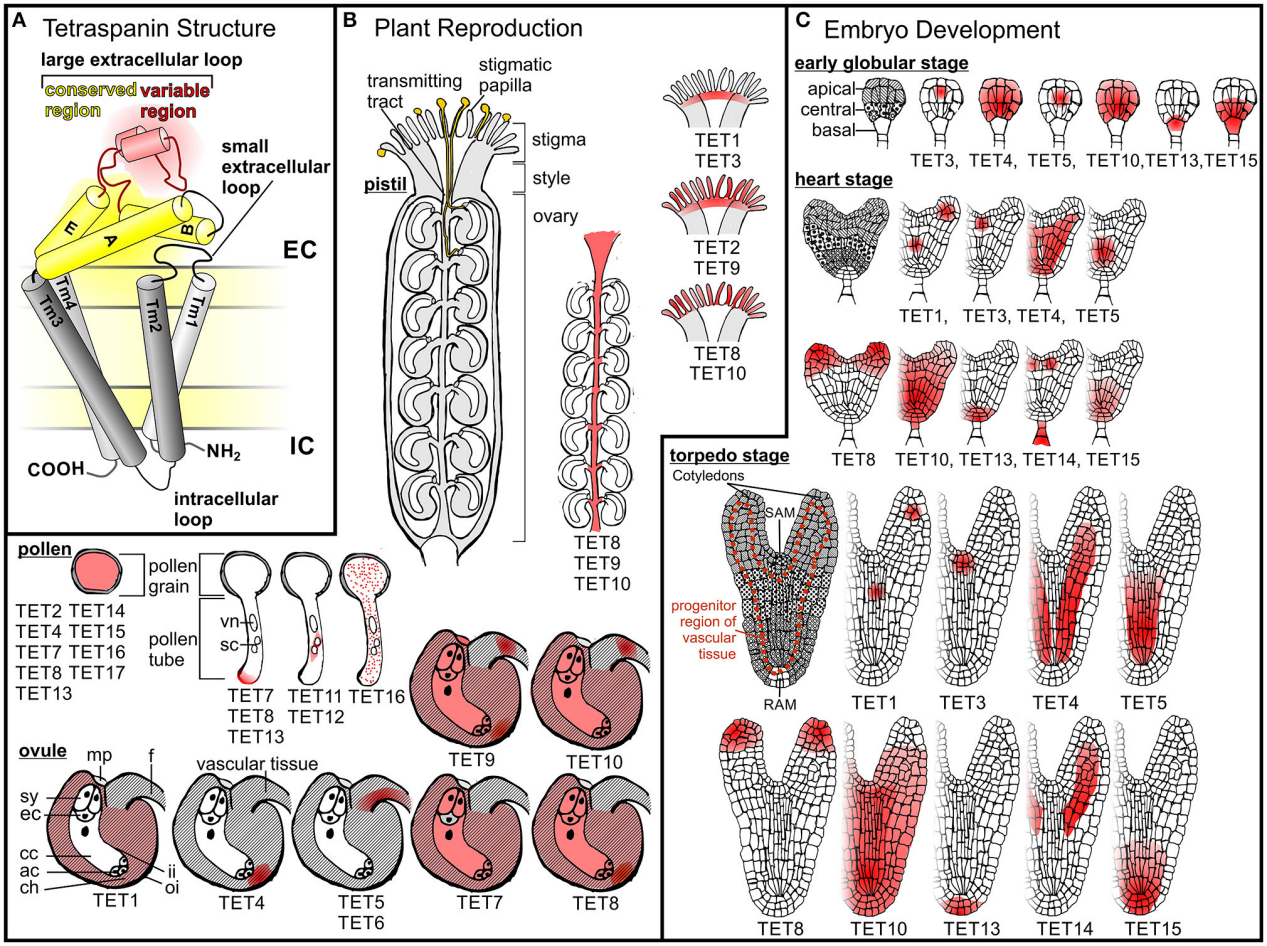
**Tetraspanin structure and expression patterns in ***A. thaliana***. (A)** A schematic representation of tetraspanin structure and membrane topology. Tetraspanins are composed of a small extracellular loop, a large extracellular loop (LEL), an intracellular loop, a N-terminal tail and a C-terminal tail. Red and yellow shading indicate the variable and conserved domains of the LEL, respectively. **(B)** Tetraspanin expression in reproductive *A. thaliana* cells and tissues. Pollen grains adhere to papilla on the stigmatic surface of the pistil, where they hydrate and subsequently germinate. Pollen tubes emerging from pollen grains grow through the transmitting tract toward the ovules, where they release their sperm cells to initiate fertilization. A germinating pollen tube representing the male gametophyte (vn, vegetative nucleus; sc sperm cells) and an ovule containing the embry sac, which represents the female gametophyte, (ac, antipodal cells; cc, central cell; ch, chalazal region of the ovule; ec, egg cell; f, funiculus; mp, micropyle; sy, synergid cells) are drawn at higher magnification. *AtTETs* expression pattern in different reproductive organs or structures as well as the subcellular localization of AtTETs in pollen tubes is indicated in red. **(C)** Tetraspanin expression pattern during *A. thaliana* embryo development. Embryos at different developmental stages are shown: globular, heart and torpedo stadium. Apical, central and basal domains are represented due to different patterns. Shoot (SAM) and root (RAM) apical meristems are indicated. *AtTETs* expression in different tissues of globular and heart shaped embryos is shown in red.

All tetraspanins have four transmembrane (TM) domains, short N- and C-terminal cytoplasmic tails, an intracellular loop as well as a small and a large extracellular loop (LEL) (Figure 1A; Hemler, 2005). The C-terminal cytoplasmic tail plays a role in sorting and intracellular targeting of tetraspanins in animals (Andreu Z, Yáñez-Mó M, 2014; Coceres et al., 2015) and is important for the function of the plant tetraspanin AtTET1/TRN2 during development (Cnops et al., 2006). The LEL contains a conserved region, probably involved in homodimerization, and a variable region, which is supposed to play a major role in interaction partner selection (Seigneuret et al., 2001; Yanez-Mo et al., 2001; Stipp et al., 2003; Seigneuret, 2006). The variable region contains a conserved motive (animals: GCC, plants: GCCK/RP) and additional conserved cytosine residues are required for the formation of disulfide bridges, which stabilize the domain structure and are important for tetraspanin function (Kitadokoro et al., 2001; Seigneuret et al., 2001; Hulme et al., 2014). Cryo-EM data (Min et al., 2006) and computational modeling (Seigneuret, 2006) suggested that TM helices form a tightly associated four-helix bundle. However, recent structural analysis of the human tetraspanin CD81 revealed that the four TM helices fold as two largely separated pairs with a cholesterol binding site in between. The cone shaped structure formed by the CD81 TMs is capped by the LEL, which appears to block access to the cholesterol binding site from the extracellular space (Zimmerman et al., 2016). Based on molecular dynamics simulations Zimmerman et al. (2016) further suggest that the LEL adopts an open conformation when cholesterol is not present in its binding site. This open conformation may promote interaction with partner proteins, whereas the closed state, when cholesterol is bound, may prevent such interactions. Besides the LEL, TM domains have been shown to be directly involved in protein—protein interactions (Kovalenko et al., 2005; Shoham et al., 2006)

Tetraspanins typically undergo post-translational modifications which affect protein stability and binding to interaction partners. Palmitoylation of intracellular cysteine residues influences protein stability (Berditchevski et al., 2002; Yang et al., 2002) and supports tetraspanin-tetraspanin interactions (Charrin et al., 2002; Yang et al., 2004), whereas glycosylation appears to be relevant for tetraspanin function (Ono et al., 2000; Baldwin et al., 2008; Wang et al., 2012) and localization (Scholz et al., 2009; Tominaga et al., 2014). In *Arabidopsis thaliana* (At) potential N-glycosylation sites in the variable region of the LEL were predicted for the tetraspanins AtTET1—4, 8, 10, 13, and 14 (Boavida et al., 2013).

## Tetraspanin interactions and dynamics

Tetraspanins associate with each other and with various integral membrane proteins such as integrins, proteins with Ig domains, membrane bound proteases and intracellular signaling molecules (Little et al., 2004; Andre et al., 2006; Le Naour et al., 2006) thereby forming distinct tetraspanin-enriched microdomains (TEMs) that function as mobile signaling hubs within membranes (Hemler, 2005; Yáñez-Mó et al., 2009). Tetraspanin interactions within TEMs occur at three distinct levels, as revealed by biochemical characterization. First level interactions are represented by primary complexes between tetraspanins and other transmembrane proteins as well as by tetraspanin homodimers or -oligomers. These interactions are direct, and resist harsh solubilization conditions (Charrin et al., 2003a; Kovalenko et al., 2004). Primary complexes are linked into a network due to the tendency of tetraspanins to form heterooligomers. Within these networks, secondary interactions occur between tetraspanin interaction partners and numerous non-tetraspanins. These interactions are indirect and much more sensitive to disruption (Charrin et al., 2003a; Hemler, 2005). The number of potential protein partners expands when third level interactions are considered. These indirect interactions can be identified in tetraspanin complexes that were isolated via cell lysis using milder (less hydrophobic) non-ionic detergents. Sucrose gradient centrifugaton results in the selective enrichment of tetraspanin complexes isolated under these conditions in the insoluble, light membrane fraction, in which lipid rafts also accumulate (Claas et al., 2001; Cherukuri et al., 2004a,b). The identification of TEMs in this membrane fraction is likely to be a consequence of palmitoylation and of tetraspanin interaction with lipids such as gangliosides and cholesterol (Hemler, 2005). The association of gangliosides or cholesterol with tetraspanins affects both, tetraspanin—tetraspanin interactions as well as the interaction of tetraspanins with other proteins (Charrin et al., 2003b; Odintsova et al., 2006; Silvie et al., 2006; Todeschini et al., 2007; Zimmerman et al., 2016). Although they share some characteritstics, TEMs and lipid rafts are clearly distinct types of microdomains as they react differently to temperature changes, cholesterol depletion or non-ionic detergents, and as most of their components do not overlap (Hemler, 2005).

More recently, different tetraspanin interaction levels were also observed and confirmed by advanced microscopy (Nydegger et al., 2006; Barreiro et al., 2008; Homsi et al., 2014; Zuidscherwoude et al., 2015). Imaging of tetraspanin dynamics revealed that these proteins are distributed throughout membranes and usually exhibiting Brownian movement during lateral diffusion. However, in some membrane areas tetraspanins remained transiently confined and accumulated (Espenel et al., 2008). The dynamics of co-diffusing tetraspanins was also observed and suggested that these proteins form mobile clusters containing other tetraspanins, partner proteins and lipids (Espenel et al., 2008; Potel et al., 2013; Zuidscherwoude et al., 2015). These small clusters may interact with each other and exchange tetraspanins indicating that protein-protein interactions within TEMs are transient and highly dynamic (Barreiro et al., 2008; Espenel et al., 2008). Furthermore it was proposed that these clusters mainly contain tetraspanin homo-oligomers and are organized as non-randomly distributed clusters of different tetraspanins adjacent to each other (Zuidscherwoude et al., 2015).

In summary, tetraspanins appear to act as dynamic master organizers within membranes, which control the distribution and clustering of associated partner-proteins and thereby regulate cellular functions, such as signaling and adhesion (Berditchevski and Odintsova, 1999; Levy and Shoham, 2005). In plants, no direct interaction partners of tetraspanins besides other family members (Boavida et al., 2013) have been identified to date. However, genetic data indicate that AtTET1 functions in a common pathway with TORNADO1 (TRN1), a leucine rich-repeat protein that regulates patterning processes during *Arabidopsis* development. Furthermore AtTET1 may function together with WINDHOSE1 and 2 (WIH1/WIH2), two small peptides, in promoting megasporogenesis (Cnops et al., 2000; Lieber et al., 2011).

## Tetraspanin mutants in plants

Although the Arabidopsis Tetraspanin (AtTET) gene family consists of 17 members and several T-DNA insertion lines have been analyzed, in which the expression of single members of this family is reduced or enhanced (Table 1), to date mutant phenotypes have been described only for *AtTET1, 13* and the functionally redundant genes *AtTET5* and *6*. Characterization of these mutants revealed an involvement of plant TETs in the control of cell proliferation, cell differentiation and cell identity, as well as tissue patterning. Such developmentally important functions have been described for tetraspanins earlier in other model organisms (Kazarov et al., 2002; Hemler, 2008; Franco et al., 2010; Anderson et al., 2011; Hou et al., 2015), suggesting that TET functions are partially conserved between kingdoms.

**Table 1:** **Overview of Arabidopsis ***Attet*** mutants and ***AtTET*** gene expression patterns described in the literature**.

**TET**	**Mutant lines**	**Embryo**	**Seedling and adult plant**	**Male tissue**	**Female tissue**	**Sources**
AtTET1	KO, KD	Provascular tissue^•^	Root◦; lateral root cap^•^; vasculature^•^; cotyledon^•^; rosette leaf^◦•^; flower^◦•^		Stigma^•^; outer integument (BP, AP)^•^	1, 2, 3, 4, 5, 6
AtTET2	KO, KD		Cotyledon^•^; rosette leaf^◦•^; flower^◦•^	Mature pollen^•^	Carpel stomata (PM)^•^; stigma^•^; papilla (BP, AP)^•^	1, 6
AtTET3	KO, KD	SAM progenitor domain^•^	Root^◦•^; quiescent center^•^; flower^◦•^		Stigma^•^	1, 6
AtTET4	KD	Central part of the embryo^•^	Root^◦•^; quiescent center^•^; flower^◦•^	Mature pollen^•^	Carpel stomata (PM)^•^; chalazal proliferating tissue^•^	1, 6
AtTET5	KO, KD	Provascular tissue^•^	Root^•^; vasculature^•^; cotyledon^•^; rosette leaf^◦•^; flower^◦•^		Vasculature in carpels and ovules^•^	1, 6
AtTET6	KD		Root^◦•^; vasculature^•^; cotyledon^•^; rosette leaf^◦•^; flower^◦•^		Vasculature in carpels and ovules^•^	1, 6
AtTET7	KO, OE		Rosette leaf^•^; flower^◦•^	Mature pollen^◦•^ and pollen tube: PM^•^ and cytoplasm^•^	Ovule: outer integument^•^ and inner integument^•^ (PM) central cell^•^; synergid (filiform apparatus)^•^; antipodals^•^	1, 3, 5, 6
AtTET8	KO, KD	Apical domain^•^, tip regions of cotyledons^•^	Root^◦•^; cotyledon^•^; rosette leaf^◦•^; flower^◦•^	Mature pollen^◦•^ and pollen tube: PM^•^ and cytoplasm (AP)^•^	Stigma (BP)^•^; papilla^•^; transmitting tract (PM) (BP, AP+)^•^; ovule: outer integument and inner integument (PM), chalazal proliferating tissue (AP)^•^	1, 3, 5, 6
AtTET9	OE		Root^◦•^; cotyledon^•^; rosette leaf^•^; flower^◦•^		Stigma (AP)^•^; papilla^•^; transmitting tract (PM, AP)^•^; ovule: (PM) upper part of funiculus^•^, ovule micropyle^•^, chalazal proliferating tissue^•^, egg cell^•^, central cell^•^, synergid^•^ antipodals^•^, endosperm (AF)^•^	1, 6
AtTET10	OE	Central part of the embryo^•^	Root^◦•^; vasculature^•^ cotyledon^•^; rosette leaf^◦•^; flower^◦•^; lateral root cap^•^		Papilla^•^, carpel valve margins (PM)^•^; stigma (BP)^•^; transmitting tract (BP, AP+)^•^; ovule: (PM) upper part of funiculus (AP), integuments and funiculus (AF)^•^	1, 6
AtTET11	KO OE		Flower^◦•^	PM and sperm cell interface^◦•^		1, 3, 5, 6
AtTET12			Root^◦•^; stipuli^•^	PM and sperm cell interface^◦•^		1, 3, 5, 6
AtTET13	KO, KD, OE	Hypophysis^•^	Quiescent center^•^; flower^◦•^	Mature pollen^◦•^ and pollen tube◦ PM^•^ and cytoplasm^•^		1, 3, 5, 6
AtTET14	KD	Provascular tissue^•^	Cotyledon^•^; rosette leaf^•^: vasculature^•^; flower^◦•^	Bicellular pollen◦: ER^•^		1, 3, 5, 6
AtTET15	OE	Basal part^•^	Root^•^; flower^◦•^	Mature pollen◦: ER^•^		1, 3, 5, 6
AtTET16	KO		Rosette leaf◦; flower^◦•^	Mature pollen^◦•^ and pollen tube◦: ER^•^		1, 3, 5, 6
AtTET17	KO		Rosette leaf◦; flower◦	Mature pollen^◦•^		1, 3, 5, 6

*KO, knock-out line; OE, overexpression line; KD, knock-down line; BP, before pollination; AP, after pollination; AF, after fertilization; PM, plasma membrane; ER, endoplasmatic reticulum; SAM, shoot apical meristem. Symbols: (+) increased expression level, (◦) RT-PCR and microarray data, (•) transcriptional and/or translational GFP and/or GUS reporter studies; (Boavida et al., 2013)^1^; (Cnops et al., 2006)^2^; (Honys and Twell, 2004)^3^; (Olmos et al., 2003)^4^; (Pina et al., 2005)^5^; (Wang et al., 2015)^6^*.

The best characterized TET in plants is AtTET1 (also referred to as TORNADO2 (TRN2) and EKEKO). Analysis of different *Attet1* mutant alleles revealed its involvement in following processes: root epidermal patterning and differentiation, establishment of leaf lamina symmetry, leaf venation patterning, (Cnops et al., 2000, 2006), controlling peripheral zone identity of the shoot apical meristem (Chiu et al., 2007) and megasporogenesis (Lieber et al., 2011).

The recent phenotypical characterization of an *Attet13* T-DNA insertion knock-out (KO) mutant indicates that AtTET13 promotes primary root growth and lateral root emergence, but restricts lateral root initiation (Wang et al., 2015). No obvious phenotype was observed in single *Attet5* KO and *Attet6* knock-down (KD) T-DNA insertion mutants. However, the *Attet5 Attet6* double mutant phenotype indicates that AtTET5 and 6 redundantly function in restricting cell proliferation during root and leaf growth (Wang et al., 2015).

Functional redundancy might also complicate the characterization of other plant TETs, as partially overlapping expression patterns have been identified for several TETs.

## TET expression patterns during plant reproduction and plant development

As only a limited set of mutant phenotypes is described, transcriptomic data and localization studies are an important source to gain insights into TET function in plants. Transcriptional analysis of *TET* expression in rice and *Arabidopsis* together with recent studies using stable transgenic plants expressing *AtTET*–reporter gene fusions constructs (Boavida et al., 2013: pAtTET::NLS3xeGFP and pAtTET::AtTET::GFP; Wang et al., 2015: pAtTET::NLS-GFP/GUS), revealed specific and partially overlapping expression patterns of *AtTET* genes during plant development and in reproductive organs (Table 1). Furthermore, observed changes in *AtTET* expression in response to developmental and environmental signals as well as the identification of regulatory elements in *AtTET* promoter regions suggest that AtTET function is highly regulated (Zimmermann et al., 2004; Winter et al., 2007; Boavida et al., 2013; Mani et al., 2015). However, attempts to find correlations between expression patterns of individual *AtTETs* and their grouping into phylogenetic clades failed, as most *AtTETs* belonging to the same clade show divergent expression patterns (Wang et al., 2015).

## TET expression in female reproductive organs

In Arabidopsis, the female reproductive organ termed pistil or carpel is composed of an ovary containing the ovules, a style, and the pollen receptive tip, the stigma (Figure 1B). *AtTETs* are expressed in all these pistil tissues. Already at the first stage of fertilization, when pollen is adhering to the papilla of the stigma, TETs seems to be involved as indicated by the expression of *AtTET2, 8, 9*–*10* in the stigmatic papilla and *AtTET1, 2, 3*, and *9* at the base of the stigma (Boavida et al., 2013). Pollen grains germinate on the stigma and form elongating pollen tubes that carry sperm cells to the ovules. On their journey through the carpel pollen tubes grow within the transmitting tract (Crawford and Yanofsky, 2008), where *AtTET8 - 10* are expressed (Boavida et al., 2013). Lured by specific signals, pollen tubes exit the transmitting tract and grow through the micropylar opening into an ovule, where they burst and release the two sperm cells contained in their cytoplasm. One sperm cell fuses with the egg cell, which gives rise to the zygote, whereas the second sperm cell fertilizes the central cell to trigger development of the endosperm (Figure 1B; Kohler and Makarevich, 2006; Ngo et al., 2007; Kanaoka and Higashiyama, 2015). Ovules are comprised of the following tissues: integuments forming the outer layers, nucellus and the embryo sac, which is embedded within the nucellus and represents the haploid female gametophyte. *AtTET* expression in the different tissues of the ovule further suggests that plant TETs also participate in ovule development, fertilization and seed development. The latter is indicated by the expression of *AtTET1, 7*–*10* in the integuments (Boavida et al., 2013), which are progenitors of the seed coat (Haughn and Chaudhury, 2005). The mature embryo sac is composed of three antipodal cells at the chalazal end, two synergids, and one egg cell at the micropylar end and two central polar nuclei, which eventually fuse to form the diploid nucleus of the central cell (Yadegari and Drews, 2004; Boavida et al., 2013; Figure 1B). Two AtTETs are present in the embryo sac: *AtTET7* and *9* are coexpressed in the synergids, central cell and antipodal cells. In addition, *AtTET9* is expressed in the egg cell. Microarray data further suggest expression of *AtTET8* in the embryo sac. Ovules and, at a later stage, developing seeds are connected to the maternal plant by the funiculus, which is responsible for nutrient supply via the vasculature (Nguyen et al., 2000; Ngo et al., 2007; Drews, 2011). In regions involved in nutrient supply of developing ovules and seeds *AtTET9* and *10* are expressed in the funiculus, *AtTET5* and *6* in the vascular tissue and *AtTET4, 8* and *9* in the chalazal region (Boavida et al., 2013). After fertilization the pistil develops into a silique, which encloses the developing seeds (Lewis et al., 2006). *AtTET10* expression was found in the valve margins, a region required for the opening of the siliques and release of the seeds.

Several *AtTETs* (*AtTET1, 2, 8*–*10*) alter their expression pattern and/or expression level upon pollination or fertilization (Table 1), which implies a diverse function of TETs in these reproductive processes. Interestingly, many of the *AtTETs* regulated upon pollination are expressed in diploid female tissues such as papilla, funiculus or transmitting tract, tissues that are involved in cell-cell communication with the male gametophyte. In addition, the observation that interactions with female tissues also influence the membrane localization of certain pollen tube AtTETs (see below) suggests a role of these proteins in crosstalk between male and female tissue.

## TET expression in the male gametophyte

During pollen development and in mature pollen, which is composed of the vegetative cell containing two sperm cells, expression of *AtTET2, 4, 7, 8, 11, 13, 15–17* was detected based on transcript level analysis and/or using transgenic lines containing reporter constructs (Honys and Twell, 2004; Pina et al., 2005; Boavida et al., 2013). Transcriptional up-regulation of several TETs in growing pollen tubes was observed using reporter gene constructs (Wang et al., 2008; Qin et al., 2009; Boavida et al., 2011) for *AtTET7, 8, and 13*. In *in-vitro* cultured pollen tubes, AtTET7 and 13 localize to the apical and subapical region of the plasma membrane as well as to cytosolic granules. Interestingly, in pollen tubes growing through female tissue AtTET7, 8, and 13 accumulate preferentially at the apical plasma membrane with about 10% of the pollen tubes showing an enriched deposition of AtTET7 at the tip. As the tip is the site of polar cell expansion specific apical membrane accumulation indicates a role of these three AtTETs in polar cell growth and/or pollen tube guidance.

Although transcriptome data suggest expression of *AtTET7, 8, 11*, and *12* in sperm cells (Borges et al., 2008), this could only be confirmed for *AtTET11* and *12* using reporter gene constructs. These two AtTETs display a very distinct localization to a membrane subdomain at the site of contact between the two sperm cells, suggesting a role in intercellular communication and/or adhesion. Interestingly, GFP-tagged AtTET9 accumulates at the same membrane subdomain when overexpressed (Sprunck et al., 2012).

Plant reproduction involves cellular processes such as cell adhesion, cell-to-cell communication and cell fusion, which have been shown to be regulated by tetraspanins in other model systems as well (Hemler, 2008; Anderson et al., 2011; Fanaei et al., 2011; Jiang et al., 2015). The expression of various *AtTETs* in the male gametophyte and female reproductive tissues, together with changes in expression patterns and levels as well as changes in protein localization upon pollination or fertilization, strongly suggest functions of plant TETs in the above mentioned processes.

## TET expression in embryos

During fertilization the egg cell fuses with one of the two sperm cells delivered by the pollen tube to form the zygote, which quickly elongates along the future apical-basal axis before undergoing its first division. Following rather regular and predictable cell divisions *Arabidopsis* embryogenesis subsequently passes through several defined stages, which are referred to as octant, globular, heart, and torpedo stage (Figure 1C; Mansfield and Briarty, 1991; Boscá et al., 2011; ten Hove et al., 2015). Analysis of stable transcriptional reporter lines by Wang et al. (2015) revealed that 9 of the 17 *AtTETs* are expressed in the early globular and heart stage embryo (Table 1). The expression patterns of these *AtTETs* suggest that they participate in defined patterning processes. For instance several *AtTETs* show specific expression in tissues involved in apical-basal patterning: *AtTET3* is expressed in the progenitor domain of the shoot apical meristem, *AtTET8* in the apical domain of heart shaped embryos and later at the tip of cotyledons, *AtTET13* in the hypophysis, the founder cell of the root meristem, and *AtTET15* in the basal part of the embryo. Functions in radial patterning during embryogenesis may be inferred from the observed specific expression of *AtTET1, 5, 10*, and *14* in vascular tissue precursor cells, and of *AtTET4* and *10* in the central part of the embryo including the progenitor region of the vascular bundle (Wang et al., 2015).

## TET expression in root and shoot

Interesting *AtTET* gene expression patterns were also found by Wang et al. (2015) in developing seedlings and adult plants. TETs are expressed in primary and secondary meristematic regions, but also in differentiated cell types, indicating that they are required for various cellular processes throughout the entire plant life (Table 1). In roots, *AtTET4* is expressed in the quiescent center (QC), *AtTET13* in the QC and surrounding stem cells, *AtTET1, 13*, and *15* in the columella, *AtTET1* and *15* in the lateral root cap, and *AtTET3* in the cortex, endodermis and pericycle. Vascular expression was shown for *AtTET5* and *6*, which restrict cell proliferation and organ growth as indicated by knock-out analysis (Wang et al., 2015), as well as for *AtTET1, 9, 14*, and *13*, which are expressed in pericycle cells and lateral root primordia. *Attet13* mutants display a weak lateral root development phenotype (Wang et al., 2015), which was suggested to be mild due to redundant functions of *AtTET3–6, 8–10*, which are co-expressed with *AtTET13* in the pericycle.

In shoots, *AtTET3* and *9* are expressed in the apical meristem (SAM), *AtTET1, 5, 6, 9, 10*, and *14* in the vascular tissue, *AtTET2, 4 and 15* in guard cells, *AtTET9* in trichomes and surrounding pavement cells, *AtTET8* and *12* in stipules and *AtTET16* at the base of flowers (Boavida et al., 2013; Wang et al., 2015). Interestingly AtTET3 localizes to plasmodesmata, suggesting an involvement in cell-to-cell communication (Fernandez-Calvino et al., 2011).

## Developmental and environmental regulation of plant TET expression

Based on their complex spatio-temporal expression patterns TETs appear to be involved in a plethora of developmental processes and to function in diverse responses to environmental signals. This view is supported e.g., by the identification of regulatory motifs in the promoter region of *TET* genes (Mani et al., 2015; Wang et al., 2015). The observations that *AtTET* expression levels and patterns are influenced by pollination and fertilization (Boavida et al., 2013), that *AtTET13* expression in pericycle cells is auxin regulated and that *AtTET8* expression is upregulated upon treatment with pathogen elicitors (Wang et al., 2015) demonstrate that *AtTETs* can be regulated by various factors.

As *cis*-elements are primarily responsible for the regulation of gene expression, the identification of such motifs helps to determine whether specific factors regulate gene expression directly or indirectly, and can contribute to an improved understanding of the divergence, overlap and redundancy in TET gene expression. Using complementary regulatory data sources and combining them Wang et al. (2015) screened for *cis*-elements in *AtTET* genes and for functional interaction with transcription factors. Based on this approach, different regulatory c*is*-elements were identified in the promoters of *AtTET1*–*6, 8, 9, 16* genes, which are consistent with the distinct expression patterns of these genes (e.g., endosperm, root, vascular tissue or pollen tube), as well as with their regulation by environmental factors (high light, cold, dehydration, drought, pathogens, sugar, abscisic acid, or brassinosteroids). In addition, a regulatory network of transcription factors and AtTET genes was described that provides a further although partial view of the transcriptional regulation of AtTETs and their position in molecular pathways during flowering time, circadian clock and defense response. This regulatory network also revealed that most *AtTET* genes are regulated by multiple transcription factors and that some duplicated *AtTET* genes shared common transcription factors (Wang et al., 2015).

## TET expression in rice

Considering the enormous potential importance of TETs in the control of plant development and in the integration of biotic and abiotic stress signaling, it appears timely to investigate this gene family in economically important crops. In *Oryza sativa* 15 *TET* genes have been annotated, which Mani et al. (2015) have recently begun to functionally characterize. Their analysis of rice microarray datasets and spatial-temporal expression profiles indicated that similar to *AtTETs OsTETs* display variable and partially overlapping expression patterns and are regulated by abiotic stresses, leaf senescence, nutrient deprivation and hormones. Furthermore, *in silico* screening for *cis*-regulatory elements in the 1 kb promoter region of *OsTETs* revealed motifs responsive to temperature (heat and cold), abscisic acid or methyl-jasmonate, as well as two motifs that confer pollen or root specific gene expression, respectively. Correlation of the data obtained based on bioinformatics with transcriptional data revealed only partial overlap suggesting a complex regulation of *OsTET* gene expression. Phylogenetic comparison of OsTETs with other plant TETs revealed that only a single family member may be monocot specific (OsTET3) (Mani et al., 2015), indicating that the majority of plant TETs have conserved functions in development and in responses to environmental signals.

## What is next in plant tetraspanin research?

The functional characterization of *tet* mutants, the detailed description of TET expression patterns at different stages during plant development, and the use of bioinformatics to predict or confirm hormonal, developmental and environmental regulation of TET expression represent an exciting tool kit to investigate TET functions in planta. Progress in this research area will heavily depend on the identification of novel mutant phenotypes, the confirmation of predictions concerning TET regulation made based on bioinformatics and on the identification of TET interaction partners. Further interesting questions to be addressed are for instance, whether plant cell membranes contain TEMs, how TET interactions modulates functions of binding partners, how lateral mobility of TETs within the membrane is regulated or whether manipulating TET expression can contribute to crop improvement. The application of novel genetic and imaging techniques in combination with (established) biochemical methods will be required to address these questions.

In plants, progress of tetraspanin research has been mainly hampered by the difficulty to identify phenotypes of single KO mutants, making it difficult to assign specific physiological function to individual TETs. To date phenotypes have been only described for the single KO mutants *Attet1* and *Attet13*, as well as for the double KO/KD mutant *Attet5 Attet6* (Cnops et al., 2000; Wang et al., 2015). The use of CRISP/Cas9 methodology promises to simplify the generation of KO mutants lacking expression of single or multiple members of the AtTET protein family. CRISP/Cas9 based gene editing will not only accelerate the identification of mutant phenotypes, but also facilitate the study of TET protein interactions, domain function, posttranscriptional modifications sites, lipid interactions etc.

With the recent publications of plant TET expression patterns and mutant phenotypes, interest in identifying direct TET interaction partners might be spurred. Despite established biochemical methods novel TET interaction partners or TEM formation have not been reported in plants. To date, the only direct TET interactions demonstrated in the literature are the formation of homo- and heterodimers by AtTET7–17, which were investigated in a heterologous expression system (Boavida et al., 2013).

Testing potential TET interactions in plant cells using live-cell imaging techniques such as bimolecular fluorescence complementation (BiFC) or Förster resonance energy transfer (FRET) can complement biochemical studies. TET membrane dynamics can be observed and quantified by single particle tracking (SPT), recovery after photobleaching (FRAP), the use of photactivatable or photoconvertible fluorescent proteins or fluorescent correlation spectroscopy (FCS). Although, TET microdomain formation might be visible as an uneven protein distribution within membranes using standard confocal microscopy, to study TET organization in detail advanced light microscopy needs to be employed. Total internal reflection fluorescence microscopy (TIRF) and super-resolution microscopic techniques such as structured illumination (SIM), stimulated emission depletion (STED), stochastical optical reconstitution (STORM), or photoactivated localization microscopy (PALM) provide enhanced resolution and are therefore suitable for this purpose.

Based on the combined pursuit of the approaches described above, exciting discoveries are expected to be made on the green side of TET research within the near future.

## Author contributions

RR, BK, and JD developed the concept of this review and wrote the manuscript.

## Funding

This work was funded by the DFG Research Training Group 1962/1, “Dynamic Interactions at Biological Membranes: From Single Molecules to Tissue.”

### Conflict of interest statement

The authors declare that the research was conducted in the absence of any commercial or financial relationships that could be construed as a potential conflict of interest.
